# Managing depression in primary care: A meta-synthesis of qualitative and quantitative research from the UK to identify barriers and facilitators

**DOI:** 10.1186/1471-2296-12-47

**Published:** 2011-06-09

**Authors:** Elizabeth A Barley, Joanna Murray, Paul Walters, André Tylee

**Affiliations:** 1 Section of Primary Care Mental Health, Health Services and Population Research Department, PO Box 28, Institute of Psychiatry, King's College London, De Crespigny Park, London, SE5 8AF, UK; 2 Section of Mental Health and Ageing, Health Services and Population Research Department, PO Box 28, Institute of Psychiatry, King's College London, De Crespigny Park, London, SE5 8AF, UK

## Abstract

**Background:**

Current management in primary care of depression, with or without comorbid physical illness, has been found to be suboptimal. We therefore conducted a systematic review to identify clinician perceived barriers to and facilitators for good depression care.

**Methods:**

We conducted a systematic literature search to identify qualitative and quantitative studies published in the UK since 2000 of GPs' and practice nurses' attitudes to the management of depression. We used principles from meta-ethnography to identify common and refuted themes across studies.

**Results:**

We identified 7 qualitative and 10 quantitative studies; none concerned depression and co-morbid physical illness of any kind. The studies of managing patients with a primary diagnosis of depression indicated that GPs and PNs are unsure of the exact nature of the relationship between mood and social problems and of their role in managing it. Among some clinicians, ambivalent attitudes to working with depressed people, a lack of confidence, the use of a limited number of management options and a belief that a diagnosis of depression is stigmatising complicate the management of depression.

**Conclusions:**

Detection and management of depression is considered complex. In particular, primary care clinicians need guidance to address the social needs of depressed patients. It is not known whether the same issues are important when managing depressed people with co-morbid physical illness.

## Background

Depression affects about 121 million people worldwide, and an estimated 5.8% of men and 9.5% of women will experience a depressive episode every year [1]. Depression is a major cause of disability and distress [2] and is expected to become the second most common cause of loss of disability-adjusted life years in the world by 2020 [3]. Rates of depression co-morbid with chronic physical illnesses such as coronary heart disease (CHD),[4-6] asthma [7-9] and rheumatoid arthritis [10] are increased compared to those in the general population [11]. When physical illness and depression co-exist, the conditions interact resulting in worse outcomes; for instance patients with CHD and depression have an approximate two-fold increase in morbidity and mortality [4-6].

In the UK 90-95% of patients with depression are treated solely in primary care [12], however, management is often suboptimal [13]. Clinical practice is likely to be influenced by clinicians' attitudes [14,15], we therefore conducted a systematic review of qualitative and quantitative studies of GPs' and PNs' attitudes to managing depression. Our aim was to identify potential barriers to and facilitators for good depression care. To do this, we used principles drawn from meta-ethnography [16] and recent guidelines for producing narrative syntheses [17] to identify common and refuted themes across studies.

## Methods

### Eligibility criteria

#### Inclusion criteria

1) Qualitative or quantitative studies containing GP or PN generated data concerning their attitudes towards and experiences of managing depression.

2) Studies published in 2000 or later. This was a pragmatic method of including a manageable number of studies and ensured we obtained data on current and relevant attitudes (2000 is after the publication of the National Service Framework for Mental Health [18]).

#### Exclusion criteria

1) Studies not conducted in UK primary care settings. This was in order to obtain attitudes relevant to primary care practice in the UK, which is where a planned new intervention will be trialled.

2) Studies focusing on a single aspect of management, e.g. antidepressant prescribing. This was because our aim was to identify broad themes which could be addressed in planned later studies specific to CHD and co-morbid depression.

3) Studies of 'psychological distress', post-natal depression, intervention studies of depression education and validation studies of attitude questionnaires. These studies were considered unlikely to provide data which could inform primary care depression management.

### Information Sources and Search

With the help of a specialist librarian, we devised a search strategy based on terms relating to depression, primary care and attitudes (Appendix 1). This was adapted for 4 databases (Medline, Embase, Psychinfo, British Nursing Index and Archives; search date 30^th ^June 2008). We also searched the reference lists of obtained papers.

### Study Selection

Titles and abstracts were screened for relevance by 1 reviewer (EB); where this was unclear, the full text was obtained. The full texts of potentially relevant articles were assessed independently by two reviewers (EB and JM). Agreement was measured using Cohen's Kappa and disagreements resolved by discussion.

### Quality assessment of included studies

Two reviewers (EB, JM) independently assessed each paper for methodological quality. For qualitative papers, the CASP checklist [19]was used. As there is no established instrument for quantitative observational studies,[20] we devised a simple checklist based on the STROBE statement [21] and a recent review of tools to assess bias in observational studies [22] (Appendix 2). Agreement was measured using Cohen's Kappa weighted for closeness of scores. Disagreements were resolved by discussion. In qualitative synthesis there is a tension between study quality and relevance,[23] so in common with other such syntheses [24,25] an inclusive approach was taken. Quality judgements were not used to exclude papers, but, the strength of findings was tested by examining whether they were supported by studies in the upper tertile of scores [25].

### Data Collection Process and Data Items

Data concerning participant characteristics, aims, setting and methods were extracted independently by two authors (JM and EB) (Additional File 1. Table 1). Two further types of data were extracted:

1. First order constructs [26]: reported attitudes and experiences of GPs and PNs (qualitative papers) and summaries of participant responses to questionnaire items (quantitative papers).

2. Second order constructs [26]: author-derived themes, conclusions, interpretations and recommendations (qualitative papers) and results headings, conclusions and recommendations (quantitative papers).

Extracted data were tabulated. The original wording or a paraphrase was used to preserve meaning [27]. The tables were examined and discussed by two authors (EB and JM) in order to ensure agreement and that second order constructs were grounded in clinician-generated data (first order constructs).

### Synthesis of Results

A grid was produced using SPSS. The rows were the included papers and the columns were second order constructs. The second order constructs were translated across studies by combining columns with broadly related headings. Two authors (EB and JM) performed reciprocal and refutational syntheses [28] to identify and summarise shared constructs across studies and constructs that were contested between or within papers. These syntheses were performed simultaneously as this is considered most informative [29]. The resulting syntheses or 'translations' were agreed by all authors through discussion. The research team included an academic GP (AT), a psychiatrist (PW) and qualitative health researcher (JM) and a health psychologist and nurse (EB).

## Results

**Figure 1:**
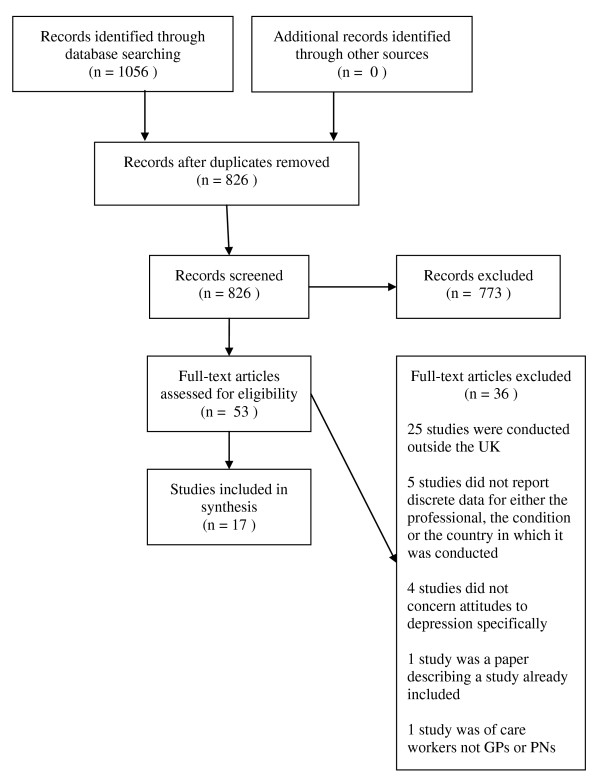
**Search results: numbers of included and excluded studies and reasons for exclusions**.

We identified 826 papers; following the initial screen, 53 were reviewed in detail. 25 were not from the UK. The remaining 28 were screened independently by 2 authors (EB and JM). 17 papers (7 qualitative and 10 quantitative) were included in the review (Cohen's Kappa = 0.68). This process and the reasons for exclusion are shown in Figure 1.

All the studies concerned adults. Four studies focused on older adults (late-life depression); we report findings separately for this population only where differences were apparent. We found no studies of managing depression co-morbid with physical illness. Characteristics of the included studies are shown in Additional File 1.Table 1. They provide data from 2,738 GPs (2,595 in quantitative studies) and 476 PNs (466 in quantitative studies) who vary in gender, age, years in practice, practice type, geographical location, level of mental health training and ethnicity. In most studies, discrete data for GPs and PNs were not reported, accordingly, in our synthesis their data are combined and identified differences highlighted.

### Quality assessment

Agreement for qualitative papers was good (weighted kappa = 0.62). All but 2 [30,31] (CASP score of 5 out of 10), were rated as of at least reasonable quality (CASP score ≥ 7). Agreement was also good for quantitative papers (weighted kappa = 0.73). We considered most of these to be low quality (achieving ≤ 4 out of 7 quality markers). They suffered from poor response rates and lack of a validated instrument to measure attitudes (Additional File 1. Table 1), few reported their selection criteria.

### Second order constructs

Most of the qualitative studies reported a simple thematic analysis and the data from the quantitative studies tended to represent similar themes. We identified 7 second order constructs; all were supported by at least one good quality study and by both qualitative and quantitative data. The identified second order constructs and supporting data from each paper are summarised in Additional File 2. Table 2. Other issues, which were identified and used to inform the synthesis, were the effects of patient gender, ethnicity and age.

#### 1.) Professionals' understanding of depression

Two contrasting understandings were identified: depression as a normal response to life events and a biomedical model of depression.

##### Depression as a normal response to life events

Problems of everyday living such as isolation, loneliness, family breakdown and lack of social support,[32-34] work stress (especially in suburban areas),[32] housing problems,[32,33] crime, unemployment and financial problems,[30,32,33] illness,[33] loss [33] and reduction in function [34] were seen as justifiably contributing to depression. This view was pronounced in studies of late-life depression where depression was attributed to the distressing effects of events associated with getting older but not to ageing per se [33,35].

Studies concerning late-life depression [33,34] suggest that clinicians working with ethnic minority patients may try to normalise depression by using words such as 'loneliness' and 'homesickness' instead of depression [34]. This may stem from clinicians' beliefs that depression is less recognised in some cultures [33]. There are insufficient data to determine if the same occurs with younger patients.

Clinicians holding a 'normalising' understanding of depression found it difficult to distinguish between distress and depression and worried about medicalising social problems [30,31,33,34,36]. Such an understanding may therefore conflict with the way in which diagnoses are made and treatments offered [31,33,34].

"If depression is conceptualized as a normal response to disadvantage, in which existential despair is the principal component, then the question of an appropriate diagnostic and management strategy could become as intractable as the illness itself"[32](p634).

##### Biomedical understandings

Depression is seen by some as 'a medical condition distinct from everyday life' [36](pe5) which is caused by neurotransmitters [36] or biochemical abnormality [35,37]. This is associated with a view that depression is not inevitable [38] and is treatable [36]. Some clinicians' encouraged patients to understand depression as biochemical even when they themselves did not hold this view [31,36]. Their aims in doing so were to:

'clarify the experience of depression, remove blame and stigma and to provide a way forward to use antidepressants'[36] (pe5).

#### 2.) Recognising depression

This construct concerned making a diagnosis, presentations of depression and the effects of a diagnosis of depression.

##### Making a diagnosis

Clinicians struggle to distinguish between 'normal' distress and depression requiring treatment [30,34,39]. Some reported using subjective processes:[30,34]

"I have my own kind of mental ways in finding out if people are depressed" [34] (p372).

Case-finding tools were criticised as excluding important external cues [30,34]. Where ratings of depression were compared with patient ratings, little agreement was found [38,40,41]. Clinician characteristics may influence recognition of depression: GPs' diagnoses were more accurate if they felt confident treating depression [38] and more recently trained nurses believed a higher proportion of their patients to be depressed [42].

Case-finding tools were not used in older people,[34] despite diagnosis in this group considered especially difficult [33,34]. Older people were perceived reluctant to accept a diagnosis of depression [34] or to talk about their mood as it would 'waste' the doctor's time [33,34]. However, such perceptions may be justification for clinicians' reluctance to make a diagnosis when they feel they have nothing to offer the patient [34].

##### Presentation of depression

Cues to depression were found to arise slowly, with patients often raising the issue when preparing to leave [39]. Younger compared with older people were perceived more willing to broach the subject, but this was only explored in studies of late-life depression [33,34]. Older people were considered more likely to attribute depression symptoms to a physical cause,[33] but, when probed, clinicians agreed that this is common in all age groups [33]. High levels of comorbidity within older people may, however, complicate depression diagnosis and lead to delay in treatment [33].

Ethnic minority (i.e. Caribbean and South Asian) elders were also thought to somatise their depression [33,34]. This was not addressed in studies concerning younger populations. Data around gender differences were conflicting. Clinicians were aware of a greater risk of suicide in men, but, where some found men less likely than women to raise psychosocial problems, others reported no differences [33].

##### Effects of a depression diagnosis

Professionals may be reluctant to diagnose depression if they feel they have nothing to offer the patient [34]. However, patients and professionals may experience secondary gain from such diagnoses [32]. For patients it may be a 'way out' of social problems or a way of avoiding work; hence GPs felt many patients seek medicalisation of their problems [32]. For the GP, giving a diagnosis of depression allows them to follow a pre-determined treatment plan and to avoid feelings of powerlessness [32].

#### 3.) Management strategies

An individualised approach based on a wide range of management options was favoured [30,34-36,43,44]. However, clinicians reported using antidepressants, psychological therapies, listening and specialist services. For nurses, the most common strategy was to make a referral to the GP [34,42].

##### Antidepressants

These were used most often [43,45]. GPs commonly considered this their only option due to a lack of availability of psychological therapy or other specialist services [31,32]. However, despite beliefs that antidepressants are effective,[35,39] it was found that prescribing guidelines were not always followed and prescriptions were for too low a dose and for too short a time [40,44,45]. Prescribing may be influenced by perceptions of patients' attitudes to antidepressants,[34,40] although GPs reported strategies to overcome negative beliefs [34]. GPs' attitudes,[38] length of experience [46] or perception of depression as moderate rather than mild [40] may also influence overall prescribing or antidepressant choice (older and more experienced GPs were more likely to prescribe tricyclics than SSRIs) [46].

In older people, uncertainty among GPs was found as to the effectiveness of antidepressants, drug interactions and side-effects [34]. There was also concern that structural factors within a practice meant that older patients on antidepressants would not be properly monitored [34]. There was a lack of data from nurses concerning antidepressant use. This may be because the nurses studied were not prescribers; it is not possible to determine this from the data.

##### Psychological therapies

Attitudes to psychological therapies tended to be positive,[32,35,39] but reports of a lack of access or availability were common [32,34,39,40,43]. One study [32] found that suburban GPs compared with inner city GPs reported greater access, but the patients may have been accessing services privately.

GPs may be less likely to refer older patients for psychological therapy, either because they 'forget' about it or assume it will not work in this population [34]. There were no data concerning ethnicity or gender in relation to psychological therapy. Data are lacking concerning nurses' views. However, in one study [42] half of the nurses reported 'counselling' patients; it is not clear what was meant by this.

##### Listening

This was considered important [30,32,36] in helping patients unburden themselves, helping clinicians uncover diverse perspectives, improving the doctor-patient relationship (by creating trust and encouraging empathy) and as a useful adjunct to antidepressants [36,39]. However, some clinicians considered their patients unable to open up [32,39] or reported an inability to empathise with a patient's chosen lifestyle [36]. Others avoided listening as they feared uncovering feelings with which they were powerless to help [34,36].

Listening requires time;[34,39,45] a lack of time was reported in several studies [31,32,34,43] but was refuted by one [39]. GPs in this study had confidence in the effectiveness of antidepressants, their skills in providing counselling support and their capacity to utilize time flexibly. This study [39] focused on time management and was able to identify more complex attitudes than the other studies. GPs may therefore be more willing or able to spend time with depressed patients than is generally thought.

##### Specialist services

Secondary care psychiatry or psychology, voluntary services and social care services were considered good quality,[43] but provision and/or access to them was commonly considered inadequate [31,34,39,43]. Lack of access to external services was seen as more of an obstacle to providing effective treatment of depression than personal knowledge or skill [43].

#### 4.) Shame and stigma

Older patients were considered more sensitive to stigma than younger patients. Older people were perceived to display embarrassment when disclosing their feelings of depression. Such feelings were hypothesised to be founded in wartime experiences where stoicism was highly prized and in 'old-fashioned' views that depression is a sign of weakness or failure to cope [33]. Fear that others may find out about their condition may be a barrier to treatment [33]. GPs were wary of using the word 'depression' with older patients in case of causing distress, but some had observed less negative reactions to questions about mood and energy [33]. However, concern about stigmatisation may be constructed to hide a reluctance to explore depression with patients arising from a desire to avoid feelings powerlessness when management options seem limited [34].

Stigma was considered more important for some ethnic groups (Caribbean and South Asian) [33]. Stigma in these communities was seen as a barrier to addressing psychosocial aspects of the illness and to beginning treatment. No study examined perceived stigma in younger people from these ethnic groups.

#### 5.) Relationships between professionals

Studies of late-life depression [33,34] indicated that GPs and PNs may have conflicting views of their roles. GPs perceived PNs as having a limited role in the identification and management of late-life depression [34]. None of the participants in one study [34] could recall a nurse referring a case to them; the GPs did not refer to nurses as they felt PNs have enough to do. In contrast, PNs saw some GPs as demotivated and unwilling to engage with depressed patients. In another study,[33] PNs felt they were in a better position to deal with depression than GPs as they had more time to explore psychosocial difficulties and operated in a less 'medical' context.

Three studies considered relationships with specialist services. One study [31] suggested that GPs' had unclear expectations of such services, another,[43] however found that GPs were satisfied by the services of specialist professionals, but complained of lack of access. PNs reported little interaction with specialist mental health services which they felt made it difficult for them to develop their knowledge and skills [42].

#### 6.) Attitudes to managing depression

Attitudes were diverse. Negative attitudes included unfavourable views of depressed people themselves e.g. 'burdens', 'not particularly attractive', 'people who bore you'[32,36], pessimism concerning outcomes [31,32,36], feelings of the work being unrewarding^19 ^and lack of confidence in their management skills especially, but not exclusively, in PNs [34,37,42,44]. Some participants were positive about the outcome of depression management [30,32,39]. However, positive attitudes may be accompanied by ambivalence, for instance some GPs were confident in managing depression, but found it 'heavy-going'[35] and required more training [46].

#### 7.) Clinicians' training needs

That GPs and PNs felt that they lacked knowledge and wanted more training was a consistent finding [32,38,42,46] This may be more common among older GPs and those without psychiatric training [46]. However, despite wanting more training, PNs did not prioritise training in mental compared with physical health [42]. A reported lack of uptake by GPs and PNs of training in the management of old age depression supports this [37].

A consistent recommendation was that training should involve consideration of professionals' views and attitudes towards depression [32,38,42] as these impact on clinical decision making [31,38]. It is also because of findings that a negative past experience of mental health training was associated with PNs' current negative attitudes towards engaging with patients' mental health needs [42].

## Discussion

This systematic review of British GPs' and PNs' attitudes did not identify any studies concerning the management of depression co-morbid with physical illness despite the common co-occurrence of mental and physical disorders [11]. The identified themes indicate barriers to and facilitators for good care in patients with a primary diagnosis of depression. Below we consider how these may relate to the care of people with depression and co-morbid physical illness.

This review indicates that depression and its diagnosis are considered complex. This is unsurprising since there is ongoing debate as to the nature of depression [47], the 'medicalisation of misery' [48] and the appropriateness of different case-finding tools [49,50] which complicates judgements about whether depression is 'under-diagnosed' or 'optimally treated' [48]. The use of case-finding tools was discussed in some of the included studies, but most of these were conducted prior to the introduction of financial incentives under the Quality and Outcomes Framework of the UK GP contract [51] in 2006 when their use became routine. A recent study [49] found, as did this review, that there is ambivalence among GPs as to their use. The detection of depression in people with physical illness, such as CHD is likely to be viewed as even more complicated given the overlap between somatic symptoms of depression and of CHD and the potential for increased anxiety in people with CHD which may also be associated with depression [52].

Management of depression is perceived as particularly complex when patients present with social problems. That GPs and PNs are aware of the relationship between social and mood problems is clear from this review, but they are unsure of its exact nature and of their role in managing it. This uncertainty may be exacerbated by a lack of attention in guidelines concerning the influence of social problems on response to treatment [53]. It may be especially important to address social problems in depressed patients where co-morbid physical illness has resulted in impaired functioning. Enhanced depression care interventions such as stepped care or collaborative care, which provide depression severity related treatment guidance to clinicians, have been shown to improve depression in chronic diseases such as diabetes and heart disease, although mortality or other disease outcomes have not improved [54-57]. However, such research has often been conducted using case detection questionnaires to identifying participants. As such, this may not reflect clinical practice where a dimensional approach to diagnosis of depression is often taken [58]. This may be particularly the case for milder forms of depression.

The other issues identified by this review, such as ambivalent attitudes to working with depressed people, a lack of confidence among some clinicians in their ability to manage this condition, the use of a limited number of management options and a belief that some patients will feel stigmatised by a diagnosis of depression also complicate the management of depression. Nevertheless, in a recent qualitative study,[59] GPs reported being able to balance a range of complex factors such as the patients' clinical presentation and motivation and their own ability to help in terms of time, skills and expertise in their decisions to refer patients for psychotherapy. It is not known whether this is the case when managing patients with depression and co-morbid physical illness.

This review has also shown that GPs and PNs vary widely in their attitudes to, confidence in and knowledge about managing depression. Most of the data is from GPs, but from the available data, PNs appear to have similar views on many issues. PNs may be less likely to manage depression than GPs, but where depression is comorbid with physical illness PNs' views may be more important since they are taking an increasing lead in chronic disease management.

### Strengths and Limitations of this review

There is no consensus concerning selection of studies for inclusion in syntheses of qualitative studies [29] or syntheses of both qualitative and quantitative studies [60]. Syntheses of qualitative studies have been conducted using a small sample of key studies [27] or the first 10 relevant papers located [61]. In common with previous work,[29] we aimed to include sufficient studies to provide a manageable quantity of rich data and as such devised a search strategy that was specific rather than overly sensitive. Since we aimed to identify broad themes, only studies which considered the whole depression management process were included. Studies which considered specific aspects, e.g. antidepressant use,[14] were excluded but may further explain the themes that we have identified.

We were interested in current experiences in the UK so we only included recent British studies, this review is therefore limited in its consideration of cross-cultural issues in managing depression. Some data concerning attitudes towards managing depression in different ethic groups was identified, but this is limited to studies of late-life depression. It is not possible to determine from this review whether this is due to a lack of emphasis on this issue in the included studies or whether the GPs and PNs studied did not consider ethnicity to be an important factor when managing depression in younger people.

A strength of this review is that the synthesis incorporates diverse perspectives from reviewers with clinical and academic knowledge of depression. This is important as syntheses of descriptive studies necessarily involve interpretation of data [23]. This contrasts with systematic reviews of treatment studies which aim to aggregate data in a way that minimises the impact of reviewer opinion (bias) [23].

'Bias' is reduced, or made explicit, in reviews of descriptive data through transparency of methodology. Here, this was achieved through the use of techniques from established qualitative synthesis methodologies such as meta-ethnography,[28] critical interpretive synthesis [29] and recent guidelines [17]. For instance whether primary study authors' interpretations (second order constructs)[26] were grounded in participant data (first order constructs)[26] was tested, primary study authors' own words were extracted [27,62] and agreements and disagreements between studies (reciprocal and refutational synthesis) were sought throughout the process [15].

In order to obtain the richest possible dataset, qualitative and quantitative studies were included. However, there is no established methodology for combining data from both types of study [60]. The comparability of studies using different methodologies was therefore tested by tabulating study type within each identified construct (Additional File 2. Table 2); this showed that each construct was supported by both study types. Use of methods from systematic reviews of treatment studies also increased the robustness of the synthesis. For instance, study selection, data extraction and quality ratings were made independently by two reviewers. Each identified construct was supported by a least one study of reasonable quality (Additional File 2. Table 2). Nevertheless, it is possible that reviewers using different methodologies may arrive at different conclusions. The explicit description of the methods employed here will help others determine where different interpretations could have been made.

## Conclusions

There is a lack of research exploring primary clinicians' attitudes to the management of depression that is co-morbid with physical illness. This review has found that British GPs and practice nurses consider the diagnosis and management of depression to be complex. In particular more guidance and support to address patients' social problems is needed, especially since mental health policy in the UK [63] promotes stronger links between health and social care. It is not known whether the same issues are important to clinicians when managing depressed people with co-morbid physical illness.

The present study was conducted as part of 'UPBEAT-UK',[64] a research programme funded for 5 years by NIHR to understand and better manage the impact of co-morbid depression on CHD in primary care. Findings of this review such as that clinicians may view and manage depression in older people differently and that certain groups are considered more likely to somatise symptoms of depression are especially relevant to the management of depression in people with CHD who are likely to be older and to have multiple physical co-morbidities. The issues raised by this review will be explored by UPBEAT-UK through qualitative studies of depressed CHD patients' and their clinicians' experience.

## Appendix 1: Medline search strategy

1. exp Depression/ or depression.mp

2. depress$.mp.

3. 1 AND 2

4. primary care.mp. OR Primary Health Care/

5. general practice.mp. OR Family Practice/

6. Health Personnel/

7. Medical staff/

8. Nurses/

9. general practitioners.mp. OR Physicians Family/

10. 4 OR 5 OR 6 OR 7 OR 8 OR 9

11. "Attitude of Health Personnel"/ OR Attitude/ or attitude.mp.

12. belief.mp.

13. perception.mp. OR Perception/

14. 11 OR 12 OR 13

15. 3 AND 10 AND 14

16. limit 15 to English language and yr "2000 - 2008"

## Appendix 2: Checklist devised for this study to assess the quality of observational studies

(answer items 1-5 'yes' or 'no')

Screening: was there a clear aim?

1.) Was the selection of participants appropriate? (consider source population, inclusion or exclusion criteria, methods of selection)

2.) Was the measurement of variables appropriate? (consider validity and reliability of instruments/measures used)

3.) Was there appropriate control of bias? (consider sources of bias, were appropriate methods outlined to deal with any issues such as recall bias, interviewer bias, non-responders, **note response rate**)

4) Was the use of statistics appropriate? (consider primary outcome stated a priori, **note sample size**)

5.) Was the study free of conflict of interest? (consider declarations of conflict of interest or identification of funding sources)

6.) list any other limitations of the study

## Competing interests

The authors declare that they have no competing interests.

## Authors' contributions

EB designed and conducted the literature search, screened papers for inclusion, critically appraised included papers, extracted data, conducted the analysis and interpretation of data and drafted the article. JM assessed the full texts of potentially relevant articles, critically appraised included papers, extracted data, conducted the analysis and interpretation of data and revised the article. PW and AT assisted in the analysis and interpretation of data and revised the article. All authors read and approved the final manuscript.

## Authors' information

EB is a practitioner health psychologist, registered general nurse, researcher and systematic review module leader for the MSc in mental health service and population research at the Institute of Psychiatry.

JM is a senior lecturer in social research specialising in qualitative studies in mental health.

PW is a research fellow and consultant psychiatrist.

AT is a GP, professor of primary care mental health and Academic Director of the Mood, Anxiety and Personality Clinical Academic Group at Kings' Health Partners, King's College London.

## Pre-publication history

The pre-publication history for this paper can be accessed here:

http://www.biomedcentral.com/1471-2296/12/47/prepub

## Supplementary Material

Additional file 1 **Table 1 characteristics of included studies**. Table 1 giving details of studies included in the review. Click here for file

Additional file 2 **Table 2 Translations of second order constructs across studies**. Table 2 describing translations of second order constructs across studies [65]. Click here for file
