# Supplementary material for: Managing depression in primary care: A meta-synthesis of qualitative and quantitative research from the UK to identify barriers and facilitators
Source: BMC Fam Pract. 2011 Jun 9;12:47. doi: 10.1186/1471-2296-12-47 (PMC3135545; doi:10.1186/1471-2296-12-47)
Supplement: Additional file 1 — Table 1 characteristics of included studies. Table 1 giving details of studies included in the review. [file 1471-2296-12-47-S1.DOC]

**Table 1.**Characteristics of included studies.

| **Reference** | **Participants**  **(RR)** | **Aim** | **Setting and sample selection** | **Methods of collecting clinician data & research perspective (quality assessment)** |
| --- | --- | --- | --- | --- |
| **qualitative studies** | | | | |
| 36 | 32 GPs  (RR 24%) | Identify issues of importance to GPs in depression management | 28 GP practices in and around Southampton (plus 2 GPs in Leicester) | semi-structured interviews; grounded theory  (10/10) |
| 33 | 18 GPs, 7 PNs | Identify perceptions of depression older people | 18 South London primary care teams in 5 Boroughs, purposive sampling based on setting (socioeconomic/ethnic groups served) and practice type | in-depth, semi-structured interviews; grounded theory  (9/10) |
| 34 | 9 GPs, 3 PNs | Explore how primary care professionals and patients view late-life depression | 1 PCT in NW England, purposive sampling, but criteria not stated | semi-structured interviews; constant comparison  (7/10) |
| 30 | 20 GPs | Explore GPs’ experience of recognizing and managing depression | 11 practices from a ‘random’ sample of 55 in Scotland – 4 NHS board areas | semi-structured interviews; critical realist perspective  (5/10) |
| 39 | 19 GPs | Investigate GP views on consultation time and depression management. | 8 West Midland practices –purposeful selection based on socioeconomic/ geographical setting and patient list size | semi-structured interviews  (8/10) |
| 32 | 35 GPs- in teaching practices  (RR ~ 66%) | Explore GP attitudes to the management of depression in deprived *vs* affluent populations | 22 inner city GPs *vs* 13 suburban and semi-rural GPs in NW England, purposive sampling based on practice size | semi-structured interviews; constant comparative qualitative analysis  (9/10) |
| 31 | 10 GPs | Explore GPs’ views on depression management | 8 practices in Greater Manchester (inner city/ suburban) | in-depth, semi-structured interviews  (5/10) |
| **quantitative studies** | | | | |
| 40 | 17 GPs | Explore associations between GP treatment, depression severity and patient characteristics | 6 GP practices in Southampton (9 practices approached) | questionnaire (devised for this study) ratings of patient characteristics and GP treatment decisions completed following consultation  (3/7) |
| 41 | 4 GPs – 3 principals, 1 assistant | Compare GPs and male patients’ assessments of depression | 1 practice in a prosperous rural area of Cheshire | questionnaire (devised for this study), completed following consultation  (3/7) |
| 42 | 442 PNs  (RR 56.2%) | Assess PNs’ knowledge, attitudes, training and management of depressed patients | 1 in 2 sample of Scottish general practices (428 Practices) | questionnaire - DAQ plus some questions developed for this study, postal survey  (5/7) |
| 45 | 202 GPs  (RR 50%) | Compare expectations of GPs and patients in the management of relapse of depression | GP practices ‘across the UK’ | questionnaire – devised for this study by a market research company, postal request sent with link to online questionnaire  (2/7) |
| 44 | 274 GPs | Describe GPs beliefs about their management of depression. | All GPs in Lambeth, Southwark and Lewisham Health Authority (inner city) | Likert scale questionnaire – (devised for this study, but piloted on 15 GPs)  (4/7) |
| 35 | 61 GPs  (RR 60%) | Assess the attitudes of GPs towards depression | Inner city GP surgeries in Lambeth (group & single-handed), ‘randomly’ selected | questionnaire (DAQ) - postal survey  (2/7) |
| 43 | 1703 GPs  (RR 48%) | Survey GPs’ perception of the availability/quality of primary care and community-based services for depressed people. Identify barriers to provision of services | 11 health authorities – 1 from each English region, and 1 each from N.Ireland, Wales and Scotland. Urban, rural, deprived and privileged | questionnaire – devised for study, piloted on 131 GPs; postal survey  (4/7) |
| 46 | 263 GPs  (RR 72%) | Examine the attitudes and practice of GPs in managing late-life depression. | All 116 practices in Nottingham Health Authority | postal survey (adapted for study from previous research) - responses to attitude statements and clinical vignettes  (4/7) |
| 38 | 40 GPs | Test hypotheses that measures of GPs’ confidence in identifying depression predict ability to identify depression and that GPs who prefer antidepressants prescribe more than those who prefer psychotherapy | practices in Liverpool and Manchester | questionnaire (DAQ), prescribing information, Likert scale depression ratings  (3/7) |
| 37 | 31 GPs, 24 PNs  (RR 12% of practices approached) | Assess acceptability and feasibility of an educational package concerning management of depression in old age | 14 practices in West Essex, East Hertfordshire, Redbridge | vignettes and questionnaire (adapted DAQ for older people) (baseline data only used in synthesis)  (4/7) |

All information was not available for each study. RR = response rate; DAQ = depression attitude questionnaire[65]; (quality assessment score: higher score = higher quality, qualitative studies scores out of 10 on CASP checklist[19];quantitative studies scores out of 7 on scale devised for this study (Appendix 2))
