# Supplementary material for: Managing depression in primary care: A meta-synthesis of qualitative and quantitative research from the UK to identify barriers and facilitators
Source: BMC Fam Pract. 2011 Jun 9;12:47. doi: 10.1186/1471-2296-12-47 (PMC3135545; doi:10.1186/1471-2296-12-47)
Supplement: Additional file 2 — Table 2 Translations of second order constructs across studies. Table 2 describing translations of second order constructs across studies [65]. [file 1471-2296-12-47-S2.DOC]

**Table 2**. Translations of second order constructs across studies

| **Summary second order constructs** | **Extracted second order constructs (authors’ ‘own words’ or paraphrase)(27)** | **Summary translation across studies** |
| --- | --- | --- |
| Professionals’ understanding of depression | ‘Constructing and resisting boundaries between depression, the self and ‘normal’ sadness’**[**36]  ‘Depression as a normal response to life events’**[**32]  Inconstistency between beliefs and practice[31]  ‘Concerns surrounding the medicalization of social problems’[30]  Depression as “understandable reaction to distressing circumstances”**[**33]  ‘Aetiology of depression’[35]  Nature of depression[35**,** 37, 38] | Depression may be seen as a normal reaction to distressing events or as pathology. Understandings of depression may conflict with management strategies. |
| Recognising depression | ‘Potential for secondary gain’**[**32]  ‘GPs’ accounts of diagnosing and caring for patients with depression’[30]  ‘GPs’ experiences of the diagnosis and management of depression’[31]  ‘consultation length/time and disclosure’**[**39]  ‘Presenting complaints’**[**33]  ‘Distinguishing between depression and physical illness’**[**32]  ‘Avoidance of psychosocial problems’**[**33]  ‘Making the diagnosis’[34]  Accuracy of diagnosis[38, 40, 41**,** 42] | Recognising depression is a complex process involving non-explicit subjective processes. Some see patients as reluctant to talk about their mood. Somatisation is common. Somatisation and/or co-morbidity may complicate diagnosis. Receiving or giving a diagnosis of depression may benefit patients and GPs. |
| Management strategies and tools | ‘GP goals and management approach’**[**36]  ‘GPs’ accounts of diagnosing and caring for patients with depression’[30]  ‘GPs experience of the diagnosis and management of depression’[31]  ‘Importance of listening’**[**36]  Time as a barrier to listening**[**32]  Time and consultation length, disclosure, antidepressants, time management**[**39]  ‘Management of late-life depression in primary care’[34]  Antidepressant use[35, 38, 40**,** 44, 45]  Role of specialist services**[**43]  Managing depression**[**42] | Clinicians used antidepressants, talking therapies, listening and specialist services. Listening to depressed patients takes time; this may be a barrier to effective treatment, but one in depth study contested this. |
| Stigma and shame | ‘Stigma and shame’**[**33]  ‘depression still carries a stigma in this age group’[34] | Depression is perceived as stigmatising for some elderly people, especially those from ethnic minority groups. |
| Relationships between professionals | ‘Primary care relationships’[34]  PNs’ position in the practice**[**33]  Confusion over the role of and lack of access to specialist services[31**, 42,** 43]  “scarcity of counselling resources”**[**39] | There is confusion between primary care staff concerning their roles and responsibilities in the diagnosis and management of depression, and about the role of specialist services which seems focused around lack of access. |
| Attitudes to managing depression | ‘GP responses to chronic depression’**[**36]  ‘Interactional difficulties with depressed people’**[**32]  Pessimistic about outcome[31]  Positive about outcome[30**,** 39]  Lack of confidence in managing depression[34, 37**, 42,** 44]  Ambivalence[35**,** 46] | GPs and PNs experience frustration in managing depression. Some are confident about outcomes, but commonly there is ambivalence. |
| Training needs | ‘without understanding the framework which underpins GPs views on ‘depression’….., educational interventions directed at GPs will not improve patient outcome’**[32]**  Lack of training and knowledge[34**,** 37**,** 42**,** 43**,** 46]  ‘education efforts should focus on increasing GPs’ sense of therapeutic optimism and providing them with sufficient skill in and knowledge of a range of psychological procedures’[38]  ‘GPs would benefit from educational programmes that promote awareness of current treatment guidelines’[45] | Many PNs and some GPs say they need more training in managing depression, but this is not a priority for them. Training should be grounded in professionals’ understandings of depression and should seek to improve attitudes to working with depressed people. |

All translated second order constructs were supported by at least one good quality study (highlighted references are studies scoring in top tertile of quality scores: qualitative studies ≥ 8/10, quantitative studies ≥ 4/7) and by both qualitative and quantitative studies
